# Endothelial-Related Gene Expression Plays a Role Against Acute Kidney Injury and Prolonged Intensive Care Stay in Liver Allografts Treated with Hypothermic Oxygenated Perfusion

**DOI:** 10.3390/medsci14010087

**Published:** 2026-02-12

**Authors:** Francesco Vasuri, Carmen Ciavarella, Giuliana Germinario, Deborah Malvi, Luca Saragoni, Antonia D’Errico, Matteo Ravaioli, Gianandrea Pasquinelli

**Affiliations:** 1 Pathology Unit, Santa Maria delle Croci Hospital, 48121 Ravenna, Italy; francesco.vasuri2@unibo.it (F.V.); luca.saragoni7@unibo.it (L.S.); 2 Department of Medical and Surgical Sciences (DIMEC), University of Bologna, 40126 Bologna, Italy; carmen.ciavarella2@unibo.it (C.C.); antonietta.derrico@unibo.it (A.D.); matteo.ravaioli6@unibo.it (M.R.); 3 Department of General Surgery and Transplantation, IRCCS Azienda Ospedaliero-Universitaria di Bologna, 40138 Bologna, Italy; giuliana.germinario2@unibo.it; 4 Pathology Unit, IRCCS Azienda Ospedaliero-Universitaria di Bologna, 40138 Bologna, Italy; deborah.malvi@aosp.bo.it

**Keywords:** gene expression, hypothermic oxygenated perfusion, liver transplantation, intensive care stay, RT-PCR

## Abstract

Background: Hypothermic oxygenated perfusion (HOPE) has emerged as a promising preservation strategy before liver transplantation, mitigating ischemia–reperfusion injury and improving graft function, especially in marginal grafts and donors after cardiac death. Methods: This is a prospective monocentric study; 34 HOPE-treated liver grafts were enrolled and analyzed through histopathology and RT-PCR to assess endothelial-related gene expression and its correlation with post-transplant outcome. The aim of the present study was to assess the relationship between the expression of genes related to vascular activation and homeostasis and post-transplant clinical characteristics. Results: Expression of SMA and TGF-β1 was significantly associated with arteriolar myointimal thickening of the graft (*p* = 0.007 and 0.068). Higher expression of SMA, ERG, and TGF-β1 was correlated with a shorter post-operative intensive care stay (*p* = 0.070, *p* = 0.010 and *p* = 0.029, respectively), particularly with post-transplant acute kidney injury. Conclusions: These findings highlight the role of endothelial activation and vascular homeostasis for an early recovery after liver transplantation, posing an important issue for healthcare systems as well, and suggesting molecular markers for graft assessment and risk stratification.

## 1. Introduction

Liver transplantation (LT) is currently the only curative option for patients with end-stage liver failure and selected liver cancers. Over the past three decades, advances in immunosuppression, surgical techniques, and perioperative critical care have substantially improved patient and graft survival. However, the persistent shortage of donor organs remains the principal obstacle to reducing mortality among individuals on the liver transplant waiting list. To meet this challenge, transplant programs are increasingly relying on marginal grafts and organs from donors after cardiac death. However, these approaches require new strategies to improve graft preservation and quality, leading to the development of liver machine perfusion as one of the most promising options. In recent years, this technique has been significantly refined and increasingly implemented as standard practice in many transplant centers [1,2,3]. In particular, hypothermic oxygenated perfusion (HOPE) has emerged as an innovative preservation technique for liver grafts, and its use has been associated with a reduced incidence of early graft dysfunction (EAD) and improved graft survival. Moreover, HOPE offers an effective means for reducing the detrimental effects of portal inflammation, thereby further enhancing post-transplant outcomes [1,2,3,4].

From a mechanistic cellular perspective, previous studies have investigated the cellular dynamics of liver graft biopsies after HOPE, exploring the specific causes of graft failure and the mechanisms by which HOPE may mitigate them, at both histological and gene expression levels. The first rodent LT models showed that HOPE protects against reperfusion injury, reducing biliary damage and preventing endothelial cell and Kupffer cell activation, with a putative effect on fibrosis prevention [2]. These results were subsequently confirmed by a porcine split-liver model as well, which showed that HOPE-perfused grafts had less inflammation, ischemia–reperfusion injury, and lower expression of cytokines, such as TNF-α, INF-γ, IL-1β, and IL-10 [5].

Our group previously analyzed the activation of the liver sinusoidal endothelial cells (LSECs) in humans using paired graft biopsies taken before and after LT; in that study, we demonstrated that LSEC function and trophism were markedly depressed during ischemia but recovered after reperfusion, with significant increases in the expression of genes involved in endothelial activation (CD34, ERG, Nestin, and VEGFR-2) [4]. Moreover, HOPE was able to enhance this recovery to significantly higher levels than static cold storage, suggesting a protective effect on microvascular homeostasis. However, the relationship between endothelial activation, vascular homeostasis, and the clinical outcome of LT recipients remains unclear.

The aim of the present study was to assess the relationship between the expression of genes related to vascular activation and homeostasis and post-LT clinical characteristics of recipients. We focused on a gene panel previously applied in a post-LT setting, critical to vascular activation; this panel includes Vascular Endothelial Growth Factor -2 (VEGFR-2), the main trigger of the neoangiogenic process [6]; the ETS-related gene (ERG), a key transcription factor involved in vascular homeostasis and endothelial integrity [7]; Nestin, a marker of progenitor cells and endothelial trophism [4]; Transforming Growth Factor (TGF-β1), a regulator of endothelial cell activation and driver of the Endothelial-to-Mesenchymal Transition (EndMT); and Smooth Muscle Actin (α-SMA), a marker of myofibroblast differentiation [8].

## 2. Materials and Methods

### 2.1. Ethics and Case Selection

This is a monocentric, prospective, and explorative study on formalin-fixed and paraffin-embedded (FFPE) tissue. The work was approved by the Ethical Committee of the Area Vasta Emilia Centrale (AVEC), protocol number 867/2022/Sper/AOUBo, date of approval 31 January 2023. All enrolled recipients received and signed an informed consent form for participation. Inclusion criteria were: (i) execution of HOPE on liver graft before LT, (ii) availability of enough FFPE tissue for analyses, (iii) age ≥18 years, and (iv) signed informed consent (recipients). All cases fulfilling these criteria in 2 years were included in the study.

The following clinical variables were collected:

- Donors: age, gender, body mass index (BMI), donor type (DBD versus DCD), cold ischemia time (CIT, in minutes), and HOPE time (in minutes).
- Recipients: age, gender, BMI, modified end-stage liver disease (MELD) score, length of hospitalization, length of post-operative intensive care (POIC), AST, ALT, bilirubin, INR for each post-surgical day from day 1 to day 7, development of early allograft dysfunction (EAD) [9], acute kidney injury (AKI) [10] and/or death.

### 2.2. Histopathological Analysis

Reperfusion (*time-zero*) surgical biopsies were taken after graft implantation in the recipients, approximately 1 h after graft reperfusion, just before closure of the recipient’s abdominal cavity, in accordance with our routine clinical practice. Biopsies were sent to our Pathology Unit, where they were sampled, fixed in formalin, and routinely processed. From FFPE blocks, 3 µm-thick sections were cut for all routine stains and RT-PCR analysis.

The following histopathological variables were collected, with the criteria previously applied in our experience [11]:

- Portal inflammation, according to Ishak’s (0–4);
- Interface hepatitis, according to Ishak’s (0–4);
- Lobular necrosis/inflammation, according to Ishak’s (0–4);
- Arteriolar myointimal thickening;
- Bile duct regression and cholestasis;
- Occurrence and percentage of microvesicular and macrovesicular steatosis;
- Lobular fibrosis;
- Portal fibrosis, according to Ishak’s.

### 2.3. RNA Extraction and Real-Time PCR Analysis

Total RNA was extracted from FFPE tissue samples using the RecoverAll Total Nucleic Acid Isolation Kit (Thermo Fisher Scientific, Carlsbad, CA, USA), according to the manufacturer’s instructions. The RNA yield and quality were measured by an ND-1000 spectrophotometer (NanoDrop, Thermo Fisher Scientific, USA). Reverse transcription was carried out in a 20 μL reaction volume using a High-Capacity Reverse Transcription Kit (Life Technologies, Carlsbad, CA, USA). For Real-Time PCR, primers specific for the target genes were designed using the NCBI BLAST tool (ver. 1.6.0) and purchased from Merck (Kenilworth, NJ, USA; Table 1). Real-Time PCR was carried out in a CFX Connect Real-Time PCR Detection System (Bio-Rad, Hercules, CA, USA) using the SYBR green mix (Bio-Rad). Target gene expression was normalized to the housekeeping gene glyceraldehyde 3-phosphate dehydrogenase (GAPDH). Data quantification was determined by the comparative 2^−ΔΔCt^ method and expressed as fold changes relative to controls.

### 2.4. Statistical Analysis

Statistical analysis was performed using Jamovi software for Windows (https://www.jamovi.org), version 2.3 (The Jamovi Project, 2022). [Computer Software]. Continuous variables are expressed as means ± standard deviations and ranges, and discrete variables as frequencies and percentages. The Spearman and Mann–Whitney tests were applied to correlate variables, followed by the Games–Howell test as post hoc validation.

A *p*-value less than or equal to 0.05 was considered significant for rejecting the null hypothesis.

## 3. Results

### 3.1. Clinical and Histopathological Data

The inclusion criteria were satisfied by 34 cases, 18 (52.9%) DBD and 16 (47.1%) DCD. All donors’ and recipients’ clinical characteristics are listed in Table 2, including the follow-up results, which recorded 10 (29.4%) cases of EAD, 6 (18.2%) cases of post-transplant AKI, and 2 (5.9%) deceased recipients. The causes of donor death were brain hemorrhage in 20 (58.8%) cases, post-anoxic encephalopathy in 9 (26.5%), head trauma in 2 (5.9%), stroke in 2 (5.9%), and not specified in 1 (2.9%). We observed a wide CIT range due to a single case of 1200 min: the mean CIT is slightly lower without this case (401 versus 424 min), without changes in the following statistics, so we did not consider it a bias. This liver graft was re-allocated twice in the Italian National Transplant network, due to the prolonged CIT. Moreover, the mean MELD score was relatively low (14.9, with a median of 13.5), but totally in line with the experience from our Institution [3,4].

Histopathological characteristics of the donor grafts after perfusion are listed in Table 3. A moderate-to-severe myointimal thickening of portal and intralobular arteries (wherever visible) was observed in 20 (58.8%) cases. Significant portal and lobular fibrosis were recorded in 18 (52.9%) and 3 (8.8%) cases, respectively.

### 3.2. Correlations Among Gene Expression, Graft’s Histopathology, and Clinical and Perfusion Data

The baseline levels of expression of some genes showed cross-correlation: in particular, SMA expression showed a positive correlation with ERG (*p* = 0.040, Spearman’s test), Nestin (*p* = 0.038), and TGF-β1 (*p* = 0.004) expression.

The only histopathological graft variable correlating with gene expression was myointimal arteriolar thickening: expression levels of SMA and TGF-β1 were significantly lower in those cases with severe arteriolar thickening, with means of 0.4 ± 0.2 and 0.7 ± 0.8 *versus* 1.9 ± 1.6 and 6.6 ± 6.7 of the cases with mild/moderate thickening (*p* = 0.007 and 0.068, respectively, Mann–Whitney non-parametric test). Statistical analyses did not reveal significant correlations among RT-PCR and donors’ and recipients’ variables, apart from the donor group (DCD versus DBD), which influenced the expression of ERG: DBD showed 0.87 ± 0.74 mean ERG expression level, while DCD showed 1.66 ± 1.16 (*p* = 0.033, Mann–Whitney test; see also Supplemental Table S1).

**Figure 1:**
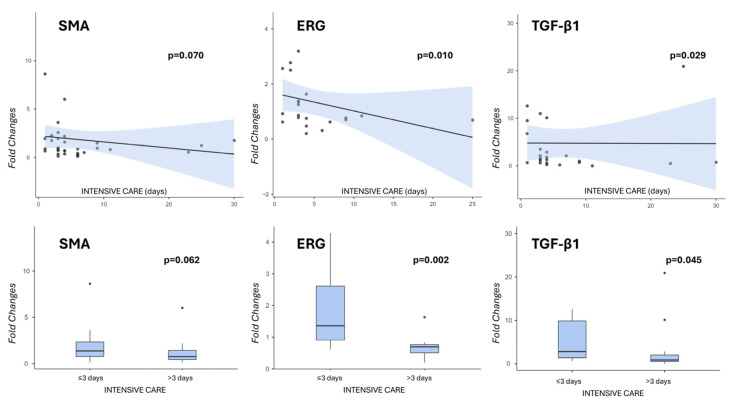
Gene expression analysis and correlation with POIC days. SMA, ERG, and TGF-b1 mRNA levels were analyzed by Real-Time PCR, showing a linearly decreased expression in recipients who stayed longer in POIC (above). GAPDH was used as a housekeeping gene, and relative mRNA expression was calculated by the 2^−∆∆CT^ method (“fold changes”). The maximum statistical significance was achieved by applying a 3-day cutoff (below).

Notably, there was a negative correlation trend between the expression of SMA, ERG and TGF-β1 and the days of POIC (*p* = 0.070, *p* = 0.010 and *p* = 0.029, respectively, according to Spearman’s test), and in particular, the expression of ERG was higher in those recipients who were discharged from intensive care within the first 3 days after LT compared to others (*p* = 0.002, Mann–Whitney test). SMA showed only a negative trend (*p* = 0.062), while TGF-β1 was significant according to the Mann–Whitney test but lost its significance upon the post hoc test (*p* = 0.203, Figure 1).

In particular, mean fold changes for ERG were 1.87 ± 1.16 in cases with POIC stay ≤3 days and 0.70 ± 0.39 in cases with POIC >3 days; mean fold changes for SMA were 2.65 ± 3.67 and 1.16 ± 1.34, respectively; mean fold changes for TGF-β1 were 6.76 ± 8.52 and 3.08 ± 5.73, respectively.

**Figure 2:**
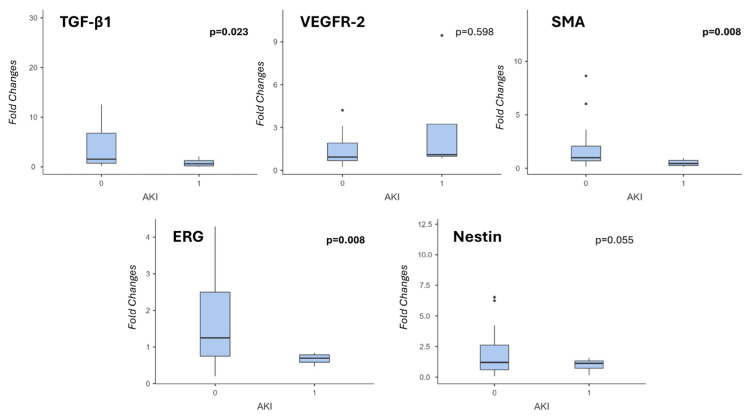
Gene expression analysis in recipients who experienced acute kidney injury (AKI). TGF-b1, VEGFR-2, SMA, ERG and NESTIN mRNA expression levels were measured by Real-Time PCR and compared between the recipients who developed acute kidney injury (AKI) after transplant and the ones who did not. Apart from VEGFR-2 (and a trend for Nestin), the expression of the analyzed genes was significantly lower in AKI patients. GAPDH was used as a housekeeping gene, and relative mRNA expression was calculated by the 2^−∆∆CT^ method (“fold changes”).

When we analyzed the recipients’ lab test in detail, in order to better understand the differences in post-transplant clinical course, we found that ERG gene expression was negatively correlated with serum AST level at post-OLT day 3 (*p* = 0.044, Spearman’s test), and Nestin gene expression was negatively correlated with total Bilirubin (*p* = 0.024). Moreover, the examined genes (except for VEGFR-2, which was not related, and Nestin, which showed a negative trend) were negatively correlated with the development of AKI in the post-transplant period, proving to be the main cause of prolonged hospitalization (Figure 2). In particular, mean TGF-β1 fold changes were 4.76 ± 7.03 and 0.85 ± 0.94 in patients who did not develop AKI versus patients who did (*p* = 0.023 Mann–Whitney test); mean SMA fold changes were 2.19 ± 3.01 and 0.50 ± 0,32, respectively (*p* = 0.008); mean ERG fold changes were 1.53 ± 1.14 and 0.67 ± 0.16, respectively (*p* = 0.008). See Supplemental Table S2 for the main histopathological characteristics in AKI and non-AKI groups.

No correlations were found between EAD and recipient mortality.

## 4. Discussion

Previous works of our group show that graft histology can affect transplant outcome in an extended criteria donor setting [12] and that HOPE is able to significantly mitigate the risk of EAD and graft loss due to histopathological risk factors, i.e., portal fibrosis and portal inflammation, when compared to grafts with similar histology but preserved with static cold storage [11]. In this prospective exploratory study, we investigated the relationship between the expression of genes related to vascular activation and tissue homeostasis in HOPE-treated liver grafts and early post-LT clinical outcomes.

Our results suggest that endothelial-related molecular signatures at the time of reperfusion reflect graft vascular integrity and are associated with short-term post-operative recovery, particularly the duration of POIC and the development of AKI. Arteriolar myointimal thickening, a well-recognized feature of chronic vascular injury, emerged as the most important histopathological graft characteristic, correlating with lower expression levels of SMA and TGF-β1. These findings support the concept that the analyzed genes mirror the microvascular adaptive potential of the graft under oxidative metabolic stress, indicating a loss of cellular trophism in livers affected by chronic vascular damage. This observation is consistent with the notion that chronic vascular injury is associated with impaired trophism and reduced adaptive remodeling capacity [4], confirming their role in tissue architecture and vascular stabilization, endothelial–mesenchymal crosstalk, and maintenance of sinusoidal architecture following injury [13,14,15].

At the molecular level, the observed correlations among SMA, ERG, Nestin, and TGF-β1 suggest a coordinated endothelial activation and repair program, as hypothesized when we chose the gene panel. ERG is a master transcription factor required for endothelial survival, nitric oxide homeostasis, and resistance to inflammatory injury [4,15], while Nestin has been described as a marker of activated endothelial cells and endothelial progenitor populations involved in vascular regeneration [4]. The co-expression of these markers in HOPE-treated grafts supports the hypothesis (including our previous experience) that HOPE preserves or restores the endothelial homeostasis, in agreement with experimental models, which demonstrated cytokine release and endothelial modulation after HOPE [3,16].

With respect to recipient follow-up, which represents the primary aim of our study, the inverse correlation between SMA, ERG, and TGF-β1 expression and the length of POIC stay is one of the most relevant findings of this study. When considered as independent variables, higher expression levels of these genes in recipients with shorter POIC stays suggest a more efficient or rapidly resolving endothelial response following reperfusion. Conversely, inefficient endothelial activation may reflect a more pronounced ischemia–reperfusion injury, requiring prolonged support. These observations are consistent with previous reports highlighting the central role of hepatic vascularization in modulating systemic inflammatory responses and hemodynamic stability after LT [17,18].

To better comprehend the reasons for the prolonged POIC stay, we further analyze the clinical and serological characteristics of recipients at different post-LT days, finding that ERG expression negatively correlated with AST levels at post-transplant day 3, further supporting the association between preserved endothelial homeostasis and improved hepatocellular integrity, inflammatory control, and tissue repair. Experimental and clinical evidence has consistently shown that sinusoidal endothelial cell injury precedes hepatocyte damage and contributes to cholestasis and parenchymal necrosis following reperfusion [19,20]. Of particular interest, we observed a negative correlation between endothelial-related gene expression and the development of post-transplant AKI. AKI remains a major determinant of morbidity and prolonged hospitalization after LT, and it is closely linked to systemic inflammation, endothelial dysfunction, and microcirculatory impairment [21,22]. One possible interpretation could be that a better-preserved graft endothelial function may attenuate systemic inflammatory signaling, thereby reducing the risk of extrahepatic organ dysfunction, as supported by studies on the so-called liver–kidney axis in the post-transplant setting [23]. However, the current study was not designed to investigate mechanistic pathways but the correlation between gene expression and clinical outcome. Future studies may benefit from expanding the gene panel and incorporating bioinformatic and systems biology approaches [24].

Interestingly, no significant correlations were observed between RT-PCR results and EAD or recipient mortality: this finding is not unexpected given the limited sample size and the multifactorial nature of EAD. Importantly, the absence of negative associations confirms once more the safety of HOPE and suggests that endothelial activation represents a protective adaptive response.

One limitation of the present study is represented by the small sample size, which, although adequate for an exploratory study, limits the statistical power and the generalization of the results. Furthermore, the monocentric design may lead to interpretative bias linked to a possible center-specific effect.

## 5. Conclusions

In conclusion, our results provide novel evidence that endothelial activation and genes related to vascular homeostasis are associated with early clinical recovery after LT, particularly with respect to intensive care duration and renal function. These results also have relevant applications for the healthcare system, given the high costs of hospitalization for transplanted patients. Our findings reinforce the central role of the hepatic microvasculature in determining LT outcomes and support further investigation of endothelial molecular markers as tools for graft assessment and risk stratification in the era of machine perfusion. Larger multicenter studies integrating molecular, functional, and clinical data are warranted to validate these observations and facilitate their translation into clinical practice.

## Figures and Tables

**Table 1:** List of primer sequences used for Real-Time PCR analysis.

Gene	Primer Sequence
ERG	*FWD* TCGCATTATGGCCAGCACTA *REV* CGTTCCGTAGGCACACTCAA
GAPDH	*FWD* AATGGGCAGCCGTTAGGAAA *REV* AGGAGAAATCGGGCCAGCTA
NESTIN	*FWD* GACCCTGAAGGGCAATCACA *REV* GGCCACATCATCTTCCACCA
α-SMA	*FWD* ACCTTTGGCTTGGCTTGTCA *REV* GGAAGCTTTAGGGTCGCTGG
TGF-β1	*FWD* TGGTGGAAACCCACAACGAA *REV* GAGCAACACGGGTTCCAGGTA
VEGFR-2	*FWD* CGGTCAACAAAGTCGGGAGA *REV* CAGTGCACCACAAAGACACG

ERG: ETS Transcription Factor; FWD: Forward; GAPDH: Glyceraldehyde 3-Phosphate Dehydrogenase; REV: Reverse; α-SMA: Smooth Muscle Actin; TGF-β1: Transforming Growth Factor; VEGFR-2: Vascular Endothelial Growth Factor Receptor 2.

**Table 2:** Baseline characteristics of donors, recipients and perfusion variables in our series (n = 34).

*DONORS*		
*Donor type*	DBD	18 (52.9%)
	DCD	16 (47.1%)
*Mean age (years)*	66.3 ± 14.3 (33–94)	
*Gender*	Females	12 (35.3%)
	Males	22 (64.7%)
*BMI*	28.0 ± 7.6 (17.5–50.6)	
*RECIPIENTS*		
*Mean age (years)*	62.0 ± 8.4 (43–75)	
*Gender*	Females	15 (44.1%)
	Males	19 (55.9%)
*BMI*	25.0 ± 4.5 (17.8–36.5)	
*MELD*	14.9 ± 7.2 (6.0–35.0)	
*Hospital stays (days)*	26.2 ± 21.6 (7–107)	
*POIC stay (days)*	6.1 ± 6.8 (1–30)	
*EAD*	10 (29.4%)	
*AKI*	6 (18.2%)	
*Deceased*	2 (5.9%)	
*Mean post-transplant AST (U/dL)*	Day 1	1099 ± 1723
	Day 2	724 ± 1100
	Day 3	389 ± 606
*Mean post-transplant ALT (U/dL)*	Day 1	561 ± 699
	Day 2	703 ± 917
	Day 3	653 ± 843
*Mean post-transplant Bilirubin (mg/dL)*	Day 1	3.90 ± 29.0
	Day 2	1.95 ± 2.65
	Day 3	2.31 ± 2.98
*Mean post-transplant INR*	Day 1	1.90 ± 0.47
	Day 2	1.47 ± 0.56
	Day 3	1.28 ± 19.62
*PERFUSION*		
*Cold ischemia time (min)*	424 ± 165 (280–1200)	
*HOPE time (min)*	146 ± 68 (65–390)	

AKI: acute kidney injury; BMI: body mass index; EAD: early allograft dysfunction; MELD: modified end-stage liver disease.

**Table 3:** Baseline histopathological characteristics of liver grafts in our series (n = 34).

*HISTOPATHOLOGICAL VARIABLE*		
*Portal inflammation (acc. Ishak)*	None-to-mild	30 (88.2%)
	Moderate	4 (11.8%)
*Interface hepatitis*	Absent	30 (88.2%)
	Present	4 (11.8%)
*Arteriolar myointimal thickening*	Absent–mild	14 (41.2%)
	Moderate	12 (35.3%)
	Severe	8 (23.5%)
*Lobular necrosis (acc. Ishak)*	Absent	14 (41.2%)
	Mild	13 (38.2%)
	Moderate	7 (20.6%)
*Lobular fibrosis*	Absent	15 (44.1%)
	Mild/focal	16 (47.1%)
	Severe/diffuse	3 (8.8%)
*Portal fibrosis (acc. Ishak)*	0	2 (5.9%)
	1	14 (41.2%)
	2	17 (50.0%)
	3	1 (2.9%)

## Data Availability

The data presented in this study are available on request from the corresponding author. The data are not publicly available due to privacy restrictions by our Ethical Committee.

## Supplementary Materials

The following supporting information can be downloaded at https://www.mdpi.com/article/10.3390/medsci14010087/s1. Supplemental Table S1. Donors’ clinical features and gene expression results between DBD (donors with brain death) and DCD (donors with cardiac death). ERG expression resulted the only variable statistically different between the two groups (Mann-Whitney test). Supplemental Table S2. Graft histology results between the cases developing post-transplant acute kidney injury (AKI) and others.

## Abbreviations

The following abbreviations are used in this manuscript:

AKI	Acute kidney injury
DBD	Donor with brain death
DCD	Donor with cardiac death
EAD	Early allograft dysfunction
FFPE	Formalin-fixed and paraffin-embedded
HOPE	Hypothermic oxygenated perfusion
LT	Liver transplantation
POIC	Post-operative intensive care
